# A Ten-Minute Bioassay to Test Metal Toxicity with the Freshwater Flagellate *Euglena agilis*

**DOI:** 10.3390/biology11111618

**Published:** 2022-11-05

**Authors:** Soyeon Choi, Hojun Lee, Min-Soo Lee, Joon Tae Park, Philippe M. Heynderickx, Di Wu, Stephen Depuydt, Jana Asselman, Colin Janssen, Donat P. Häder, Taejun Han, Jihae Park

**Affiliations:** 1 Department of Marine Science, Incheon National University, 119 Academy-ro, Incheon 22012, Korea; 2 Bio Environmental Science and Technology (BEST) Laboratory, Ghent University Global Campus, 119-5 Songdomunhwa-ro, Incheon 21985, Korea; 3 Department of Biology, Incheon National University, 119 Academy-ro, Incheon 22012, Korea; 4 Division of Life Sciences, College of Life Sciences and Bioengineering, Incheon National University, Incheon 22012, Korea; 5 Center for Environmental and Energy Research, Ghent University Global Campus, 119-5 Songdomunhwa-ro, Incheon 21985, Korea; 6 Department of Green Chemistry and Technology, Faculty of Bioscience Engineering, Ghent University, 653 Coupure Links, B-9000 Ghent, Belgium; 7 Laboratory of Plant Growth Analysis, Ghent University Global Campus, 119-5 Songdomunhwa-ro, Incheon 21985, Korea; 8 Department of Applied Ecology and Environmental Biology, Ghent University, Coupure Links 653-Block F, B-9000 Ghent, Belgium; 9 Department für Biologie, Friedrich-Alexander University, Staudtstr. 5, 91058 Erlangen, Germany; 10 Department of Animal Sciences and Aquatic Ecology, Ghent University, Coupure Links 653-Block F, B-9000 Ghent, Belgium

**Keywords:** bioassays, *Euglena agilis*, end points, heavy metals

## Abstract

**Simple Summary:**

Bioassays can offset the limitations of traditional chemical analyses (time constraints, high cost, and limited detection of interactions) in monitoring water pollution. *Euglena*, a flagellate green alga, is an attractive experimental model organism that has been used for toxicity testing for decades because it is easy to culture, grows rapidly, and responds quickly to environmental stresses. In the present study, we examined the effects of seven heavy metals in the native Korean *E. agilis* using six end points (motility, velocity, cell compactness, upward swimming, *r*-value, and orientation). The advantage of the ecotoxicity assay presented here is its rapidity. Unlike the usual 3–4 d of exposure time and work, this assay takes only 10 min to obtain results; moreover, it can be performed at room temperature under dark conditions. Therefore, this new method can be useful for the rapid toxicity screening of hazardous pollutants, as it may have operational advantages over test time.

**Abstract:**

A chemical analysis of water quality cannot detect some toxicants due to time constraints, high costs, and limited interactions for detection. Bioassays would offer a complementary means to assess pollution levels in water. *Euglena* is a flagellate green alga and an excellent system for toxicity testing thanks to its ease of culture, rapid growth, and quick response to environmental stresses. Herein, we examined the sensitivity of *E. agilis* to seven heavy metals by analyzing six end-point parameters: motility, velocity, cell compactness, upward swimming, *r*-value, and alignment. Notably, the velocity of *E. agilis* was most sensitive to cadmium (96.28 mg·L^−1^), copper (6.51 mg·L^−1^), manganese (103.28 mg·L^−1^), lead (78.04 mg·L^−1^), and zinc (101.90 mg·L^−1^), while *r*-values were most sensitive to arsenic (12.84 mg·L^−1^) and mercury (4.26 mg·L^−1^). In this study, velocity and *r*-values are presented as useful biomarkers for the assessment of metal toxicity in *Euglena*. The metals As, Cd, Cu, and Pb were suitable for this test. The advantages of the ecotoxicity test are its rapidity: It takes 10 min to obtain results, as opposed to the typical 3–4 d of exposure time with intensive labor. Moreover, this test can be performed at room temperature under dark conditions.

## 1. Introduction

The development of industrial and agricultural activities concomitant with the increase in human population has led to metal pollution in the environment, with elevated concentrations of metals such as arsenic (As), cadmium (Cd), copper (Cu), lead (Pb), manganese (Mn), mercury (Hg), and zinc (Zn) polluting aquatic ecosystems [1,2]. Metals are significant components of wastewater and industrial waste that remain in the environment after release and can accumulate in the food chain and harm organisms at various trophic levels [1]. 

Aquatic environments are generally thought to be resilient, providing a convenient and safe location for the disposal of wastes from a range of industrial and municipal origins; however, to maintain the integrity of aquatic systems, environmental protection strategies including effective monitoring and regulation need to be developed. Traditionally, water quality has been estimated by chemical analysis employing sophisticated equipment, but this approach is time-consuming and particularly limited when using mixtures of chemicals whose interactions are just as important as individual pollutants [3]. A significant limitation of relying solely on chemical analyses is that it is not possible to analyze all potential toxicants in the sample. Additionally, chemical monitoring in most environmental studies does not necessarily reflect toxicity toward living organisms [4,5]. To circumvent these limitations, bioassay-based toxicity testing methods are being increasingly used to complement chemical monitoring using a range of microorganisms, invertebrates, fish, and algae [6,7].

As primary producers, algae form the basis of aquatic food webs, and any impact on their health can disrupt the population dynamics and productivity of the entire system. Algae are easily exposed to toxic contaminants in water and reach equilibrium with pollutants quickly because they are small and have a relatively large surface area [8]. The adverse effects of metals on algal metabolism have been extensively documented. In general, exposure to metals inhibits algal growth, photosynthesis, and spore production; suppresses cell division; causes the loss of photosynthetic pigments; and damages membranes [9]. Since any adverse effect on algae can trigger damage to the entire ecosystem via trophic chains, the early and rapid toxicity assessment of water contaminated with heavy metals is important to minimize environmental damage and reduce the threat to ecosystems.

*Euglena* is one of the widely used organisms for the biomonitoring of water pollutants. It is a unicellular, motile microalga belonging to the phylum Euglenophyta and is found in many aquatic freshwater habitats [10]. The flagellate exhibits characteristic behavior controlled by gravity and light to achieve the optimal position in the water column for growth, photosynthesis, and survival [11,12,13,14,15]. *Euglena* possesses both plant- and animal-like characteristics and reproduces via asexual cell division, which improves its genetic stability [16]. Numerous behavioral, biochemical, morphological, and physiological parameters have been investigated in ecotoxicological studies of *Euglena* because of its rapid and sensitive response to various toxic substances such as heavy metals and organic and inorganic compounds [17,18].

End-point assays such as cell growth and photosynthesis have been used in numerous studies to assess the effects of various substances on *Euglena* [19,20,21,22,23]. Motility, orientation, and morphological parameters such as percentage motility, swimming speed, upward swimming, and cell shape have also been widely used to assess the effects of external stressors such as heavy metals, organic and inorganic pollutants, increased salinity, and ultraviolet radiation in this alga [14,17].

Conventional microalgal bioassays use growth measurements of cell number, dry biomass, and optical density as end points, which require relatively long exposure times, typically in the range of 72 to 96 h [24,25,26]. Such long time scales make microalgal-based bioassays unsuitable as efficient tools for routine water quality monitoring. Accordingly, faster methods have emerged to counter these limitations, such as evaluating the inhibition of algal photosynthetic activity by determining phosphate uptake, ^14^C assimilation, chlorophyll a fluorescence, and dissolved oxygen, with the aim of reducing exposure times to less than 24 h while maintaining good test sensitivity [27,28,29,30,31,32]. In this context, it is worth noting that the precision of gravitactic orientation in *E. gracilis* was a more sensitive parameter for effluent toxicity in short-term tests (immediately after exposure) than other common bioassays such as the algal growth test, the *Daphnia* motility test, the fish mortality test, and the bacterial bioluminescence test (MICROTOX) [33].

In this study, we investigated the effects of short-term (10 min) metal exposure on the movement parameters of *Euglena agilis* Carter. We compared acute toxicity thresholds obtained from algal movement patterns (ECOTOX biosystem) to those from other end-point assays in *Euglena* species to determine whether the rapid *E. agilis*-based bioassay would be an ideal system for the ecotoxicological assessment of metals in aquatic environments.

## 2. Materials and Methods

### 2.1. Algal Test Species and Culture Conditions

*Euglena agilis* Carter was cultured in mineral medium (pH 5; Checcucci, et al. [34]) in 1-L Erlenmeyer flasks at 25 °C under white fluorescent light (FL400, Kum-Ho, Seoul, Korea) with 30 μmol photons m^−2^·s^−1^ of photosynthetically active radiation (PAR; 400–700 nm) in a 16 h light/8 h dark photoperiod. All experiments were performed with exponentially growing cells under these conditions.

### 2.2. Testing Chemicals and Exposure

Heavy metals (CAS numbers 7440-38-2 [As], 7440-43-9 [Cd], 7440-50-8 [Cu], 7439-97-6 [Hg], 9439-96-5 [Mn], 7430-92-1 [Pb], and 7440-66-6 [Zn]) purchased from Sigma Aldrich (Saint Louis, MO, USA) and Junsei Chemicals (Tokyo, Japan) were used to prepare test solutions at the desired concentrations by serial dilution from stock. Toxicity tests of 10 min in duration under dark conditions were performed in a custom-made chamber consisting of two sandwiched glass slides (75 × 25 × 1.1 mm, Marienfeld, Germany) with a test volume of 70 μL of metal stock solution and 10 μL of *Euglena* cell suspension to obtain final concentrations ranging from 0.01 to 100 mg·L^−1^. In the case of untreated controls, the metal stock solution was replaced with the solvent. The initial cell density was 10.5 × 10^5^ cells mL^−1^. All treatments were performed in triplicate.

### 2.3. Measurement of Motility and Orientation

**Figure 1:**
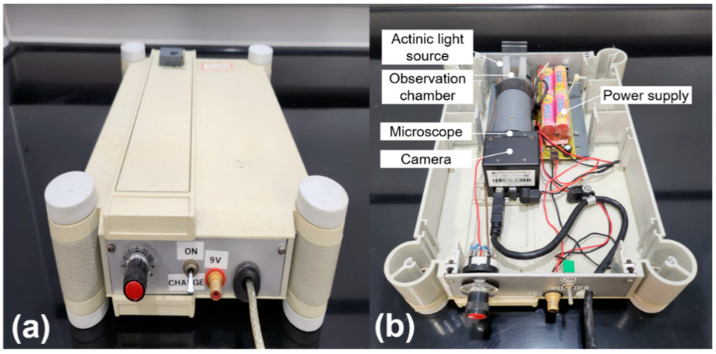
Overview of the Ecotox equipment (**a**) and the main hardware elements (**b**): The *Euglena* cell suspension is filled into an observation chamber consisting of two separate slides and inserted into a slot at the top of the device.

A horizontal custom-built microscope with a 6.3× objective was used to monitor the swimming behavior of the cells in a hand-made observation cuvette with an inner length of 10 mm and a width of 1 mm. Cells were observed under an infrared diode (λ = 875 nm) to prevent phototactic movement and the stimulation of photosynthetic oxygen production by exposure to visible light wavelengths during observation. A minimum velocity of 5 μm·s^−1^ was manually set to avoid measuring sinking and immobile objects. The vectors of the tracks were used to calculate the percentage of moving cells (motility) and their mean velocity using the software supplied with the ECOTOX biosystem (Figure 1; Tahedl and Häder [10]). The velocity of moving cells was recorded in μm·s^−1^.

The percent motility of cells was calculated by Equation (1).

(1) $$\mathrm{Motility}=\frac{n_{s}}{n}\cdot 100\%,$$

where *n* is the number of all calculated vectors, and *n_s_* the number of vectors with a velocity higher than a predefined threshold value.

The velocity in µm·s^−1^ was calculated using Equation (2).

(2) $$\mathrm{Velocity}=\frac{d}{\Delta t}\cdot ƒs,$$

where *d* = ((Δx^2^) + (Δy^2^))^0.5^; Δx and Δy are the distances in the x and y directions, respectively; Δt is the time delay between the first and fifth frame (160 ms) in the video sequence; and ƒ*s* is a scaling factor.

The cell compactness of the form factor (which is the ratio between the circumference and the area normalized to a circle) was calculated by the software using Equation (3). A cell compactness has the lowest value of 1 when the outline of the object is a circle and increases as the cell increases in length.

(3) $$\mathrm{Compactness}=\frac{\sum_{i=1}^{n_{s}}\frac{S_{i}^{2}}{A_{i}\cdot 4\pi }}{n_{s}},$$

where *S* is the length of the outline; *A* is the area of the object; and *n_s_* is the number of all calculated vectors.

Upward swimming was calculated by the system using Equation (4).

(4) $$\mathrm{Upward}=\frac{n_{0}}{n_{s}}\cdot 100\%,$$

where *n*_0_ is the number of vectors with −90 degrees ≤ *α* < 90 degrees, and *n_s_* is the total number of cells. The upward direction is defined as 0 degrees.

The *r*-value is calculated according to Equation (5).

(5) $$r-\mathrm{value}=\frac{\sqrt{{\left(\sum_{i=1}^{n_{s}}\sin\alpha_{i}\right)}^{2}+{\left(\sum_{i=1}^{n_{s}}\cos\alpha_{i}\right)}^{2}}}{n_{s}},$$

where *α* is the angle of the movement vector, and *n* is the number of all calculated vectors.

The alignment was calculated using Equation (6).

(6) $$\mathrm{Alignment}=\frac{\sum_{i=1}^{n_{s}}\left|\sin\alpha_{i}\right|-\sum_{i=1}^{n_{s}}\left|\cos\alpha_{i}\right|}{n_{s}},$$

### 2.4. Statistical Analyses

Data presented in this report are means ±95% confidence intervals (Cis). All parameters were compared between treatments using one-way analysis of variance (ANOVA) (*n* = 3; *p* < 0.05). Multiple least significant difference (LSD) comparison tests were then performed to detect significant differences between samples and controls and between treatments. The effective concentration at which 50% inhibition occurred (EC_50_) was estimated using the linear interpolation method (ToxCalc 5.0, Tidepool Science, Palo Alto, CA, USA). The coefficient of variation (CV), the standard deviation as a percentage of the mean, was calculated to estimate the precision of the tests.

To evaluate sensitivity (i.e., the lower the EC_50_ value, the more sensitive the end point) and reliability (i.e., the lower the amplitude of the coefficient of variation, the more reliable the end point), the EC_50_ values and CV of all end points were ranked in descending order regardless of metals, and then scores from 1 to 42 (six end points and seven metals) were assigned to the two sequences. In the case of an identical sequence, a mean value was assigned to end points that had the same values.

## 3. Results and Discussion

After the exposure of *E. agilis* to seven metals for only 10 min, all measured parameters significantly decreased. The metal sensitivity based on the EC_50_ values decreased in the order Hg > Cu > As > Pb > Mn > Cd > Zn (Table 1). Cu, Mn, and Zn are essential metals for algae, while As, Cd, Hg, and Pb have no known biological function in algae, although Cd could play a role as a co-factor in carbonic anhydrase and compensate for Zn deficiency in diatoms [35].

The motility parameter indicates the percentage of cells moving at a speed equal to or faster than the minimum velocity set in the program. The number of motile cells of *E. agilis* decreased when exposed to each of the seven metal species. The EC_50_ values of the inhibition of motility for the most and least toxic metals were 7.93 mg·L^–1^ for Hg and 170.99 mg·L^−1^ for Zn (Table 1).

In addition to the reduction in motility, the velocity of *E. agilis* cells also diminished, with EC_50_ values of 6.43 mg·L^−1^ for the most toxic metal, Hg, and 101.90 mg·L^−1^ for the least toxic metal, Zn (Table 1). Velocity indicates the mean velocity (swimming speed) of all moving cells. Stallwitz and Häder [36] studied the effect of heavy metals on the motility of *E. gracilis* and established that exposure to 0.6 mg·L^−1^ Hg for more than 4 d resulted in a significant reduction in cell motility and velocity. The sensitivity of‘ *E. agilis* to Hg measured here was about 10 times lower than that of *E. gracilis*, but the exposure time in our study was also much shorter (10 min) than in the study by Stallwitz and Häder [36] (5760 min). Metal exposure slowed down the swimming speed of *E. agilis* and was then followed by a reduction in motility. The higher sensitivity of swimming speed over motility has been previously reported in *E. gracilis* exposed to pollutants such as fertilizers, heavy metals, and herbicides [10,18,23].

Motility is a fundamental property that enables an organism to move in a coordinated manner in a search for food or a suitable ecological niche for survival and growth [37]. The reduction of the motility and speed of flagellate cells may limit their ability to adapt to a changing environment. Therefore, *E. agilis* cells damaged by metal exposure would be subjected to unfavorable living conditions, such as excessive or insufficient light intensities, resulting in reduced growth and photosynthesis.

The cell compactness of the form factor is the ratio between the circumference and the area normalized to a circle and describes the shape of a cell. Cell compactness has the lowest possible value of 1 when the outline of the object is a perfect circle and increases as the cell increases in length and deviates from a circle. The EC_50_ values of the inhibition of compactness for the most and least toxic metals were 6.50 mg·L^−1^ for Hg and 158.48 mg·L^−1^ for Zn (Table 1). After exposure to metals, there was a significant reduction in cell compactness, indicating that the cells took on a more spherical shape. Species of the genus *Euglena* have been reported to change their cell shape in rapid response to increasing physical or chemical stressors [38,39,40], and this phenomenon can be used as a rapid indicator for the toxic effects of pollutants [23].

Upward swimming gives the percentage of cells that are swimming towards the upper part of the cuvette (±90 degrees around the vertical direction upward); we measured EC_50_ values of the inhibition of the velocity of 5.16 mg·L^−1^ for Hg and 145.50 mg·L^−1^ for Zn (Table 1). The *r*-value describes the precision of the gravitactic orientation of swimming cells and ranges from 0 (when the cells are moving randomly) to 1 (when all cells are moving in the same direction). In this study, the *r*-value presented EC_50_ values of 4.26 mg·L^−1^ for Hg and 129.16 mg·L^−1^ for Zn (Table 1). Alignment is used to describe whether the movement of cells is horizontal or vertical; its value ranges from −1 (when there is movement of cells only in the horizontal direction) to 1 (when there is movement of cells only in the vertical direction). The EC_50_ values for alignment were 7.46 mg·L^−1^ for Hg and 169.61 mg·L^−1^ for Zn (Table 1). These three end points are all related to the gravitactic orientation of swimming cells, and all responded in a similar manner to metal exposure.

*Euglena* cells show a gravity-mediated response called gravitaxis and either swim towards gravity (positive gravitaxis) or away from gravity (negative gravitaxis) [36]. Positive gravitaxis leads an organism down into the water column, while negative gravitaxis brings it to the surface. In *Euglena*, the precision of gravitaxis is regulated by an internal rhythm determined by the daily light–dark cycle [41]. In case of *E. gracilis*, cells have been reported to exhibit negative gravitaxis in natural habitats but show a clear transition from positive to negative gravitaxis under laboratory conditions as the culture grows from exponential to stationary phase [36]. *E. agilis* was also shown to display gravitaxis, with movement patterns influenced by stress factors such as ultraviolet-B (UV-B) radiation and phenol [40,42]. Considering that gravity is an important cue for choosing a niche in the environment [41], the random vertical movement and orientation after metal exposure could lead to *E. agilis* not being able to find optimal conditions for survival.

**Figure 2:**
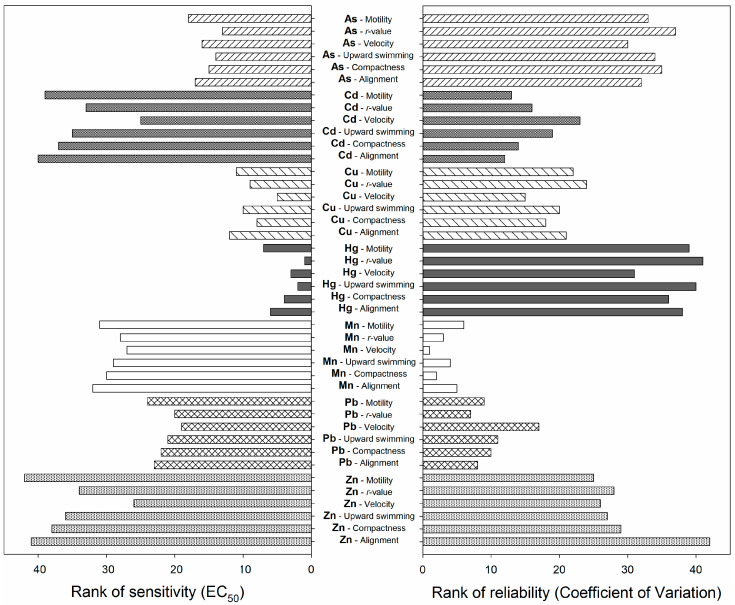
Mean ranks of sensitivity and reliability for each of the six end points (motility, *r*-value, velocity, upward swimming, compactness, and orientation) in *Euglena agilis* tests with seven metals (As, Cd, Cu, Hg, Mn, Pb, and Zn).

In this study, the most sensitive end point for the tested metals was velocity for Cd (96.28 mg·L^−1^), Cu (6.51 mg·L^−1^), Mn (103.28 mg·L^−1^), Pb (78.04 mg·L^−1^), and Zn (101.90 mg·L^−1^) and the *r*-value for As (12.84 mg·L^−1^) and Hg (4.26 mg·L^−1^). The least sensitive end point was motility for As (23.58 mg·L^−1^), Hg (7.93 mg·L^−1^), Mn (107.46 mg·L^−1^), Pb (90.27 mg·L^−1^), and Zn (170.99 mg·L^−1^) and alignment for Cd (161.68 mg·L^−1^) and Cu (9.72 mg·L^−1^) (Figure 2). As shown in Figure 2, the decreasing order of metal sensitivity of the end-point group was Hg > Cu > As > Pb > Mn > Cd > Zn. When we examined the reliability of the end-point group, the reliable data were those obtained in the order Mn > Pb > Cd > Cu > Zn > As > Hg. The appropriate sensitivity and reliability of the end points tested with *E. agilis* could therefore be ensured when testing the toxicity of As, Cd, Cu, and Pb. Previous studies on the effects of metal toxicity in *Euglena* have used a metal exposure period ranging from 1 h to 120 d at a temperature of 20–28 °C and various light conditions ranging from complete darkness to continuous illumination. The associated end-point values were based on chlorophyll a fluorescence, chlorophyll contents, delayed fluorescence, DNA damage, growth, photosynthesis, and reactive oxygen species formation (Table S1; [10,17,19,20,43,44,45,46,47,48,49,50,51,52,53,54]). In this study, the EC_50_ values based on velocity and *r*-value in *E. agilis* appeared to be within the EC_50_ range reported in other end-point studies (Table S1). Moreover, in the related species *E. gracilis*, the sensitivity of motility parameters to various toxicants is similar to other commonly used bioassays with *Daphnia* (motility), fish (mortality), algae (growth rate), and *Vibrio fischeri* (bioluminescence assay) [10,17]. Considering that only 10 min are needed for a complete measurement of velocity and *r*-value of an *E. agilis* sample together with the appropriate control, the method presented here would be an ideal system for ecotoxicological assessments of metals in the aquatic environment.

Our literature survey revealed that there is only one test method that requires a shorter exposure time (3 min) than ours (10 min). However, considering that the criteria for selecting an appropriate ecotoxicity test include sensitivity, simplicity, and cost-effectiveness, the current 10 min method is far superior to the 3 min method: our method is much more sensitive to metals such as Cd, Cu, and Hg (Table S1) than the 3 min method. The 3 min method uses a large automatic instrument that is set up in a fixed location, while our instrument is portable and small, so that it can be used outside the laboratory.

## 4. Conclusions

The ultimate goal of bioassay testing is to provide representative and comprehensive criteria for water or sample quality. To ensure a thorough assessment of the risks posed by contaminants to the environment and human health, the test methods should be sensitive, simple, and ecologically relevant [55].

In ecotoxicology, the time at which toxicity is assessed is a critical factor, and a test method should quickly provide toxicity results for management decisions. In the present study, we observed significant changes in velocity and gravitational orientation for *E. agilis* cultures after a metal exposure of only 10 min, in contrast to other studies that employed exposure times ranging from 1 h to 120 d (Table S1). Furthermore, as shown in Table S1, the proposed end points showed higher sensitivity than some other end points previously reported with *Euglena*.

Microalgae play a central role in the primary production of aquatic ecosystems. Moreover, the ingestion of microalgae in polluted waters is the main route by which toxic chemicals enter the food chain. The results of the present study confirm that metals have phytotoxic effects on *E. agilis* by exerting stress on locomotor traits. Severe locomotion impairment leads to a partial loss of physiological and ecological competence and threatens the survival of the entire population.

In this respect, a test assessing the speed and gravitational orientation of *E. agilis* would be a valuable tool, as toxicity can be determined rapidly and easily without losing sensitivity, promoting timely management decisions, especially in the event of unexpected pollution incidents.

## Figures and Tables

**Table 1:** EC_50_ values (mg L^−1^) of seven metals with different end points in *Euglena agilis*.

End Points	As	Cd	Cu	Hg	Mn	Pb	Zn
Motility	23.58	161.37	9.36	7.93	107.46	90.27	170.99
*r*-value	12.84	122.6	8.91	4.26	105.09	84.86	129.16
Velocity	21.44	96.28	6.51	6.43	103.28	78.04	101.90
Upward swimming	19.68	134.08	9.31	5.16	106.24	86.05	145.50
Compactness	21.41	155.42	8.81	6.50	106.99	88.57	158.48
Alignment	22.58	161.68	9.72	7.46	107.50	90.08	169.61

## Data Availability

All the results found are available in this manuscript.

## Supplementary Materials

The following supporting information can be downloaded at: https://www.mdpi.com/article/10.3390/biology11111618/s1, Table S1: List of metal toxicity tests with different end points in *Euglena* spp.
