# Supplementary material for: A Ten-Minute Bioassay to Test Metal Toxicity with the Freshwater Flagellate Euglena agilis
Source: Biology (Basel). 2022 Nov 5;11(11):1618. doi: 10.3390/biology11111618 (PMC9687118; doi:10.3390/biology11111618)
Supplement: Supplementary file 1 [file biology-11-01618-s001.zip › biology-1983029-supplementary.pdf]

Table S1. List of metal toxicity tests with different end points in *Euglena* spp.

Toxi-cants	End points	Test species	Tempera-ture (℃)	Photon flux density (μmol photons $m^{-2}s^{-1}$)	Light period (L:D)	Growth medium	Exposure duration	EC_{50} (mg L^{-1})	References
As	Motility	*E. agilis*	20-25	-	Dark	Mineral medium	10 min	23.58	**This study**
		E. gracilis	20-24	95	24:0	Organic medium	7 d	>10	[43]
	r-value	*E. agilis*	20-25	-	Dark	Mineral medium	10 min	12.84	**This study**
		E. gracilis	20-24	95	24:0	Organic medium	7 d	>10	[43]
	Velocity	*E. agilis*	20-25	-	Dark	Mineral medium	10 min	21.44	**This study**
		E. gracilis	20-24	95	24:0	Organic medium	7 d	>10	[43]
	Upward swimming	*E. agilis*	20-25	-	Dark	Mineral medium	10 min	19.68	**This study**
	Compact	*E. agilis*	20-25	-	Dark	Mineral medium	10min	21.41	**This study**
		E. gracilis	19	92	24:0	Mineral medium	3min	141	[10,44]
	Alignment	*E. agilis*	20-25	-	Dark	Mineral medium	10 min	22.58	**This study**
	Growth	*E. gracilis*	20-24	95	24:0	Organic medium	7 d	0.5	[43]
Cd	Motility	*E. agilis*	20-25	-	Dark	Mineral medium	10 min	161.37	**This study**
		E. gracilis	22	92	24:0	Complex and Mineral medium	3 min	202.7	[19,44]
		E. gracilis	22	92	24:0	Complex and Mineral medium	24 h	0.86	
	r-value	*E. agilis*	20-25	-	Dark	Mineral medium	10 min	122.6	**This study**
		E. gracilis	22	92	24:0	Mineral medium	3 min	183.8	[45]
		E. gracilis	22	92	24:0	Mineral medium	24 h	6.06	
		E. gracilis	22	92	24:0	Complex and Mineral medium	3 min	199	[19,44]
		E. gracilis	22	92	24:0	Complex and Mineral medium	24 h	6	
	Velocity	*E. agilis*	20-25	-	Dark	Mineral medium	10 min	96.28	**This study**
		E. gracilis	22	92	24:0	Complex and Mineral medium	3 min	500	[19,44]
		E. gracilis	22	92	24:0	Complex and Mineral medium	24 h	N.D.	
	Upward swimming	*E. agilis*	20-25	-	Dark	Mineral medium	10 min	134.08	**This study**
		E. gracilis	22	92	24:0	Complex and Mineral medium	3 min	405	[19,44]
		E. gracilis	22	92	24:0	Complex and Mineral medium	24 h	15	
	Compact	*E. agilis*	20-25	-	Dark	Mineral medium	10 min	155.42	**This study**
	Alignment	*E. agilis*	20-25	-	Dark	Mineral medium	10 min	161.68	**This study**
	Chl-con-tents	*E. gracilis*	20-25	22	12:12	Hutner medium	7 d	91.66	[46]
		E. gracilis	22-27	79	12:12	Hutner medium	10d	>9.17	[47]
	DNA dam-age	*E. gracilis*	25	-	-	Hutner medium	5 d	1.15, ca.	[48]
	Growth	*E. gracilis*	24-28	-	24:0	Cramer-Myer's medium	5 d	0.18	[20]
		E. gracilis	20-25	-	Dark	Hutner medium	7 d	1.83, ca.	[46]
			-	-	Dark	Mineral medium	7 d	>366.64	[49]
		E. gracilis	22-27	79	12:12	Hutner medium	10 d	18.33	[47]
	Mortality	*E. gracilis*	22	92	24:0	Mineral medium	3 min	202.7	[45]

		E. gracilis	22	92	24:0	Mineral medium	1 d	0.86	
		E. gracilis	-	-	Dark	Mineral medium	7 d	183.32	[49]
	ROS	*E. gracilis*	25	-	-	Hutner medium	1 h	18.33	[48]
Cu	Motility	*E. agilis*	20-25	-	Dark	Mineral medium	10 min	9.36	**This study**
		E. gracilis	22	92	24:0	Complex and Mineral medium	3 min	19	[19,44]
		E. gracilis	22	95	24:0	Mineral medium	5 d	19.09	[50]
		E. gracilis	22	92	24:0	Complex and Mineral medium	24 h	23.4	[19,44]
	r-value	*E. agilis*	20-25	-	Dark	Mineral medium	10 min	8.91	**This study**
		E. gracilis	19	83	24:0	Mineral medium	3 min	8	[14]
		E. gracilis	22	92	24:0	Complex and Mineral medium	3 min	20.4	[19,44]
		E. gracilis	22	92	24:0	Complex and Mineral medium	24 h	61	
		E. gracilis	22	95	24:0	Mineral medium	5 d	20.4	[50]
	Velocity	*E. agilis*	20-25	-	Dark	Mineral medium	10 min	6.51	**This study**
		E. gracilis	22	92	24:0	Complex and Mineral medium	3 min	23	[19,44]
		E. gracilis	22	92	24:0	Complex and Mineral medium	24 h	63	
		E. gracilis	22	95	24:0	Mineral medium	5 d	23.09	[50]
	Upward swimming	*E. agilis*	20-25	-	Dark	Mineral medium	10 min	9.31	**This study**
		E. gracilis	22	92	24:0	Complex and Mineral medium	3 min	50	[19,44]
		E. gracilis	22	92	24:0	Complex and Mineral medium	24h	52	
		E. gracilis	22	95	24:0	Mineral medium	5 d	50	[50]
	Compact-ness	*E. agilis*	20-25	-	Dark	Mineral medium	10 min	8.81	**This study**
		E. gracilis	19	92	24:0	Mineral medium	3 min	4.0	[10,44]
	Alignment	*E. agilis*	20-25	-	Dark	Mineral medium	10 min	9.72	**This study**
	Delayed fluores-cence	*E. gracilis*	20	95	24:0	Mineral medium	3 h	286.5	[51]
	Growth	*E. gracilis*	25-27	300	14:10	Mineral medium	7 d	7.98	[52]
		E. gracilis	25	28	12:12	Taub and Dol-lar's salt solution	-	>15.96	[53]
	Mortality	*E. gracilis*	20-25	-	24:0	-	3 d	28.23	[54]
	Photosyn-thesis	*E. gracilis*	25-27	300	14:10	Mineral medium	7 d	7.98	[52]
	Photosyn-thesis fluo-rescence	*E. gracilis*	20	95	24:0	Mineral medium	3 h	491	[51]
Hg	Motility	*E. agilis*	20-25	-	Dark	Mineral medium	10 min	7.93	**This study**
		E. gracilis	22	92	24:0	Complex and Mineral medium	3 min	38	[19,44]
		E. gracilis	22	92	24:0	Complex and Mineral medium	24 h	35	
	r-value	*E. agilis*	20-25	-	Dark	Mineral medium	10 min	4.26	**This study**
		E. gracilis	22	92	24:0	Complex and Mineral medium	3 min	51	[19,44]

		E. gracilis	22	92	24:0	Complex and Mineral medium	24 h	26.3	
	Velocity	*E. agilis*	20-25	-	Dark	Mineral medium	10 min	6.43	**This study**
		E. gracilis	22	92	24:0	Complex and Mineral medium	3 min	50	[19,44]
		E. gracilis	22	92	24:0	Complex and Mineral medium	24 h	38.7	
	Upward swimming	*E. agilis*	20-25	-	Dark	Mineral medium	10 min	5.16	**This study**
		E. gracilis	22	92	24:0	Complex and Mineral medium	24 h	39.8	[19,44]
		E. gracilis	22	92	24:0	Complex and Mineral medium	3 min	59	
	Compact-ness	*E. agilis*	20-25	-	Dark	Mineral medium	10 min	6.5	**This study**
	Alignment	*E. agilis*	20-25	-	Dark	Mineral medium	10 min	7.46	**This study**
	Speed	*E. gracilis*	19	83	24:0	Mineral medium	3 min	1	[17,44]
	Chl-con-tents	*E. gracilis*	22-27	79	12:12	Hutner medium	10 d	>1.36	[47]
Mn	Motility	*E. agilis*	20-25	-	Dark	Mineral medium	10 min	107.46	**This study**
	r-value	*E. agilis*	20-25	-	Dark	Mineral medium	10 min	105.09	**This study**
	Velocity	*E. agilis*	20-25	-	Dark	Mineral medium	10 min	103.28	**This study**
	Upward swimming	*E. agilis*	20-25	-	Dark	Mineral medium	10 min	106.24	**This study**
	Compact-ness	*E. agilis*	20-25	-	Dark	Mineral medium	10 min	106.99	**This study**
	Alignment	*E. agilis*	20-25	-	Dark	Mineral medium	10 min	107.5	**This study**
Pb	Motility	*E. agilis*	20-25	-	Dark	Mineral medium	10 min	90.27	**This study**
		E. gracilis	19	92	24:0	Mineral medium	3 min	40.1	[10,44]
	r-value	*E. agilis*	20-25	-	Dark	Mineral medium	10 min	84.86	**This study**
	Velocity	*E. agilis*	20-25	-	Dark	Mineral medium	10 min	78.04	**This study**
	Upward swimming	*E. agilis*	20-25	-	Dark	Mineral medium	10 min	86.05	**This study**
	Compact-ness	*E. agilis*	20-25	-	Dark	Mineral medium	10 min	88.57	**This study**
	Alignment	*E. agilis*	20-25	-	Dark	Mineral medium	10 min	90.08	**This study**
	Chl-con-tents	*E. gracilis*	22-27	79	12:12	Hutner medium	10 d	>33.12	[47]
Zn	Motility	*E. agilis*	20-25	-	Dark	Mineral medium	10 min	170.99	**This study**
		E. gracilis	19	92	24:0	Mineral medium	3 min	164	[10,44]
	r-value	*E. agilis*	20-25	-	Dark	Mineral medium	10 min	129.16	**This study**
	Velocity	*E. agilis*	20-25	-	Dark	Mineral medium	10 min	101.9	**This study**
	Upward swimming	*E. agilis*	20-25	-	Dark	Mineral medium	10 min	145.5	**This study**
	Compact-ness	*E. agilis*	20-25	-	Dark	Mineral medium	10 min	158.48	**This study**
	Alignment	*E. agilis*	20-25	-	Dark	Mineral medium	10 min	169.61	**This study**
	Mortality	*E. gracilis*	20-25	-	24:0	-	3 d	119.93	[54]

* Chl-contents; Chlorophyll-contents, ROS; Reactive oxygen species
